# Population Structure and Genetic Diversity of Shanlan Landrace Rice for GWAS of Cooking and Eating Quality Traits

**DOI:** 10.3390/ijms25063469

**Published:** 2024-03-19

**Authors:** Lin Zhang, Bowen Deng, Yi Peng, Yan Gao, Yaqi Hu, Jinsong Bao

**Affiliations:** 1 Key Laboratory of Nuclear Agricultural Sciences of Ministry of Agriculture and Zhejiang Province, Institute of Nuclear Agricultural Sciences, College of Agriculture and Biotechnology, Zijingang Campus, Zhejiang University, Hangzhou 310058, China; dalin@zju.edu.cn (L.Z.); 22216121@zju.edu.cn (Y.P.);; 2 Hainan Institute, Zhejiang University, Yazhou Bay Science and Technology City, Yazhou District, Sanya 572025, China

**Keywords:** Shanlan rice, cooking and eating quality, genome-wide association study, waxy

## Abstract

The Shanlan landrace rice in Hainan Province, China, is a unique upland rice germplasm that holds significant value as a genetic resource for rice breeding. However, its genetic diversity and its usefulness in rice breeding have not been fully explored. In this study, a total of eighty-four Shanlan rice, three typical japonica rice cultivars, and three typical indica rice cultivars were subjected to resequencing of their genomes. As a result, 11.2 million high-quality single nucleotide polymorphisms (SNPs) and 1.6 million insertion/deletions (InDels) were detected. Population structure analysis showed all the rice accessions could be divided into three main groups, i.e., Geng/*japonica* 1 (GJ1), GJ2, and Xian/*indica* (XI). However, the GJ1 group only had seven accessions including three typical japonica cultivars, indicating that most Shanlan landrace rice are different from the modern japonica rice. Principal component analysis (PCA) showed that the first three principal components explained 60.7% of the genetic variation. Wide genetic diversity in starch physicochemical parameters, such as apparent amylose content (AAC), pasting viscosity, texture properties, thermal properties, and retrogradation representing the cooking and eating quality was also revealed among all accessions. The genome-wide association study (GWAS) for these traits was conducted and identified 32 marker trait associations in the entire population. Notably, the well-known gene *Waxy* (*Wx*) was identified for AAC, breakdown viscosity, and gumminess of the gel texture, and *SSIIa* was identified for percentage of retrogradation and peak gelatinization temperature. Upon further analysis of nucleotide diversity in *Wx*, six different alleles, *wx*, *Wx^a^*, *Wx^b^*, *Wx^in^*, *Wx^la/mw^*, and *Wx^lv^* in Shanlan landrace rice were identified, indicating rich gene resources in Shanlan rice for quality rice breeding. These findings are expected to contribute to the development of new rice with premium quality.

## 1. Introduction

Rice (*Oryza sativa* L.) remains the paramount food crop worldwide, sustaining over half of the global population. As the population continues to grow, the requirement for rice production escalates correspondingly. On the other hand, there is an ascending demand for high-yield and superior-quality rice and a more affluent lifestyle. As a material cultural heritage in Hainan, Shanlan landrace rice represents the rich farming and living history of the Li ethnic people in Hainan Province, China, who have developed rice cultivars with distinctive agronomic characteristics and strong drought resistance through traditional slash-and-burn cultivation in the mountains for more than a thousand years [1]. Previous studies have found that most Shanlan landrace rice belong to the Geng/*japonica* subspecies [2,3]. Yang et al. [4] conducted a study on the genetic diversity using simple sequence repeat (SSR) markers in 57 Shanlan rice accessions and found that the subspecies *indica* and *japonica* were mixed in most clades. Li et al. [5] utilized SSR markers to analyze the genetic diversity of 214 upland rice accessions from five provinces (regions) in Southeast Asia and South China and suggested that the Shanlan rice in Hainan may have originated from Guangdong Province. Previous studies on Shanlan rice have primarily concentrated on drought resistance, while less attention has been paid to the grain quality traits.

Enhancing rice grain quality has become a top priority in rice breeding programs. To achieve this, a comprehensive understanding of the genetic control of rice cooking and eating qualities (CEQs) are imperative for improving rice grain quality [6]. The starch physicochemical properties, i.e., amylose content (AC), gel consistency (GC), gelatinization temperature (GT), and pasting viscosity, are important indices for evaluating rice CEQs. Knowledge of genetic factors underling the quality traits is required for the development of rice varieties with excellent CEQs and a genome-wide association study (GWAS) is one of the promising approaches for discovering such associated genetic factors. Previous studies have identified major genes responsible for the formation of CEQs, which are considered the most complex quantitative traits in rice. AC is the most important factor that affects the CEQs of milled rice [6]. Amylose is synthesized by the action of GBSSI in the rice endosperm, encoded by the *Waxy* gene (*Wx*). Various natural allelic variations in *Wx* including *wx*, *Wx*^a^, *Wx*^in^, *Wx*^b^, *Wx*^mw/la^, *Wx*^mp^, *Wx*^mq^, *Wx*^op^, *Wx*^/hp^, and *Wx*^lv^ have been discovered, which correspond to the diverse apparent amylose content (AAC) among rice germplasm [7,8,9,10,11,12,13,14,15,16,17]. The *Wx* gene is also responsible for the genetic basis of starch properties, such as gel consistency, pasting viscosity, and retrogradation properties [17,18,19,20,21,22,23,24]. Soluble starch synthase *IIa* (*SSIIa*) is currently the only major gene known to be associated with GT [6]. Tian et al. [24] found that AAC negatively correlated with GC and GT, and GC positively correlated with GT. In addition, other genes, especially the starch-synthesis-related genes also have minor effects on rice CEQs [25,26].

With the development of high-throughput sequencing and other biotechnological tools, the GWAS has been verified to be a useful approach for identifying genes, alleles, or haplotypes related to any traits of interest under complex environments. The GWAS has played a pivotal role in identifying many important genes which govern various complex agronomic traits such as flowering time, plant height, and panicle length in rice [27,28,29,30,31,32], and particularly starch-related parameters [20,33,34]. However, GWAS analysis for the CEQs traits has not been conducted in the Shanlan landrace rice.

The objectives of this study are as follows: (1) to dissect the population structure of Shanlan landrace rice by a next-generation resequencing approach; (2) to evaluate the genetic diversity in the physical and chemical properties of Shanlan rice and conduct a GWAS for the quality traits; (3) to identify novel alleles for the quality traits. The results obtained will provide valuable insights for quality breeding of Shanlan landrace rice.

## 2. Results

### 2.1. Identification of SNPs and InDels in Shanlan Landrace Rice by Next-Generation Sequencing

The diverse collection of ninety rice accessions is comprised of eighty-four Shanlan landrace rice (84), three typical japonica rice cultivars, i.e., Nipponbare (Nip), Zhonghua 11 (ZH11), and Tainung 67 (TN67), and three typical indica rice cultivars, i.e., 9311, Guangluai4 (GLA4), and IR36 (Table S1 in the Supporting Information). The Illumina HiSeq X10 platform was applied to acquire the pair-ends of 150 bp reads. Short reads were mapped to the Nipponbare reference genome (MSU version 7.0). After SNP calling, we identified a total of 11.2 million SNPs in which 2,619,968, 1,725,345, 224,337, and 2,399,456 SNPs were located within exons, introns, UTRs, and intergenic regions, respectively. These SNPs were further classified as 1,412,309 non-synonymous, 12,979 splice site, 77,011 stop-gain, and 4254 stop-loss in coding regions. Meanwhile, we identified a total of 1.6 million high-quality insertion/deletions (InDels). We found 1,635,574, 158,603, 379,545, and 93,626 InDels located within exons, introns, UTRs, and intergenic regions, respectively, including 1971 splicing InDels, 6580 stop-gain, and 343 stop-loss InDels in coding regions. After filtering for missing data less than 0.2 and minor allele frequency greater than 0.05, we selected 3,425,822 SNPs for use in subsequent analyses (Table 1).

### 2.2. Genetic Population Structure

**Figure 1:**
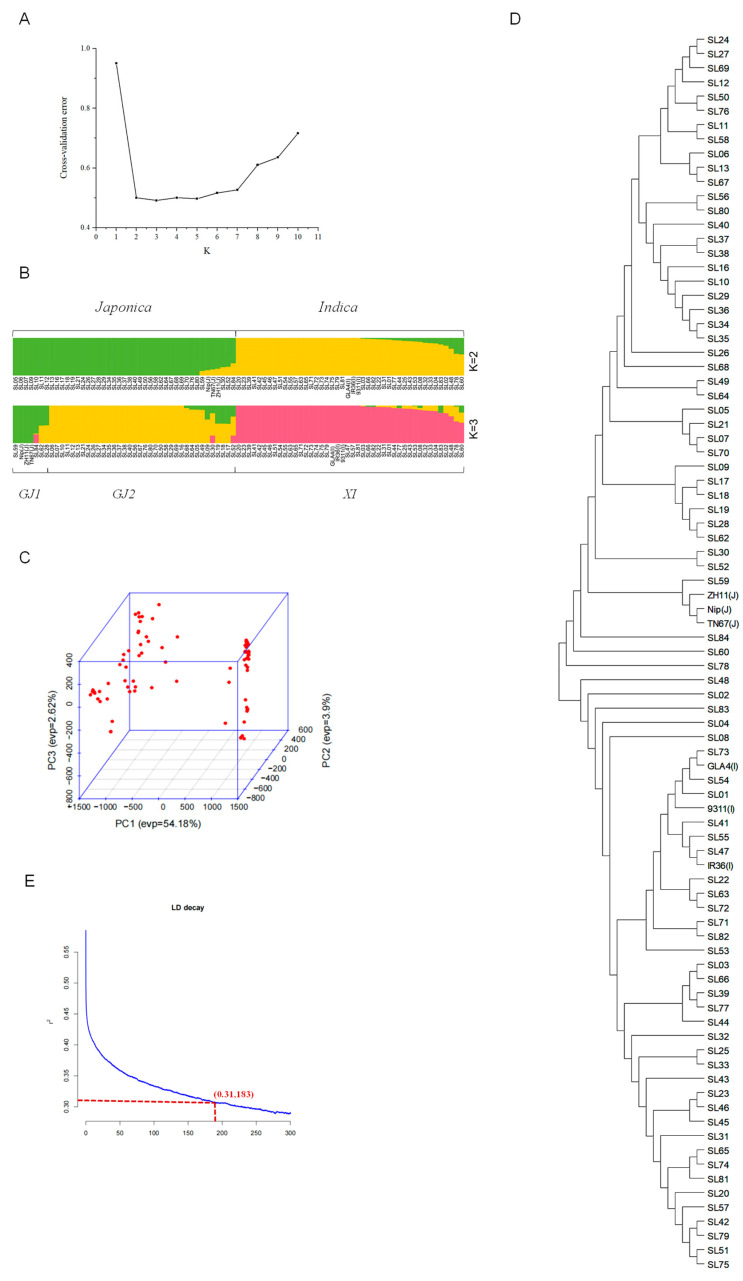
Population structure and genetic diversity in 90 rice germplasms. (**A**) Cross-validation error for K= 2–10 from ADMIXTURE analysis showing that the panel had 3 groups. (**B**) Population structure plot (K = 2, 3). (**C**) PCA plot of the first three principal components. (**D**) Phylogenetic tree based on genetic distance. (**E**) Genome-wide average LD map of all accessions.

The rice accessions are mainly divided into two distinct populations, Geng/*japonica* (GJ) and Xian/*indica* (XI). Population structure analysis showed the ideal K value with the least cross-validation error detected by the population structure analysis was determined as 3 (Figure 1A), so that the whole panel could be clustered into three main groups, named GJ1, GJ2, and XI, respectively (Figure 1B). When K = 2, as expected, all rice accessions could be divided into the *japonica* and *indica* subspecies. However, the GJ1 group only had seven accessions, including SL28, SL59, SL62, SL84, and three typical *japonica* rice cultivars. All the indica accessions remained as the XI group either selecting K = 2 or K = 3. Principal component analysis (PCA) showed that the first three principal components explained 60.7% of the genetic variation, and the germplasms in the GJ2 subpopulation were clustered significantly closer together than those in the GJ1 subpopulation (Figure 1C). The phylogenetic tree showed that the GJ1 and GJ2 subpopulations had significant genetic differentiation, consistent with the results of the population structure and PCA (Figure 1D). Linkage disequilibrium (LD) analysis showed that the LD attenuation distances of all the germplasms were 183 kb (Figure 1E). The Fst value between the GJ1 and GJ2 populations was 0.1881, indicating that there was a large genetic differentiation between the two populations.

### 2.3. Phenotypic Variation in the Study Population

**Figure 2:**
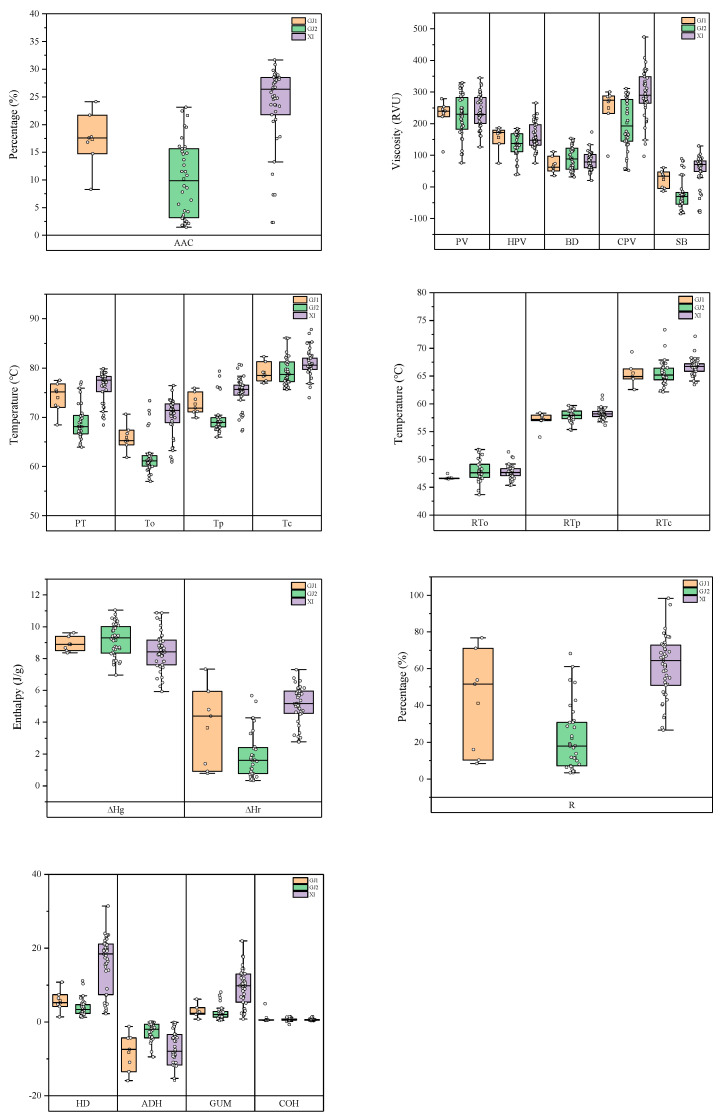
The distributions of quality traits in three panels, the GJ1, GJ2, and XI panels. PV: peak viscosity; HPV: hot paste viscosity; CPV: cold paste viscosity; BD: breakdown; SB: setback; PT: pasting temperature; AAC: apparent amylose content; HD: hardness; ADH: adhesiveness; GUM: gumminess; COH: cohesiveness; To: onset temperature; Tp: peak temperature; Tc: end temperature; ΔHg: enthalpy change; RTo: retrogradation onset temperature; RTp: retrogradation peak temperature; RTc: retrogradation end temperature; ΔHr: retrogradation enthalpy change; R: percentage of retrogradation.

The starch physicochemical properties including the parameters of AAC and pasting viscosity, texture parameters, thermal properties, and retrogradation properties were evaluated in rice accessions (Figure 2). The mean values of AAC were 16.4%, 8.9%, and 23.5% in three panels, respectively. The differences for AAC were significant between panels. XI exhibited a broader range in AAC, hot paste viscosity (HPV), cold paste viscosity (CPV), breakdown (BD), and setback (SB) compared to the other groups. Remarkably, its mid-range values were significantly higher for AAC, CPV, and SB. GJ2 had the lowest average AAC, SB, and pasting temperature (PT). However, there was no significant difference in PV, HPV, or BD among the groups. Furthermore, the retrogradation peak temperature (RTp) values of GJ2 were significantly higher than those of GJ1. For the textural parameters, the differences of most of parameters in the three panels were significant between subpopulations, except for the hardness (HD), gumminess (GUM), and cohesiveness (COH). There are obvious differences in the thermal properties of the materials, but the differences in degradation properties are relatively insignificant. These results suggest that these rice accessions are representative in terms of rice starch physicochemical properties and are qualified for further genetic analysis.

A cluster analysis was conducted based on the quality traits of the entire rice population, and a dendrogram was established as depicted in Figure S1. At a distance of 10, all the rice accessions tested could be categorized into three major groups. Glutinous rice accessions were classified into the first group. The second group, which contained the largest number of rice accessions, comprised accessions with diverse AAC values. Notably, SL04 was categorized into a separate group. This accession exhibited high viscosity and a low gelatinization temperature, which was different from the others, suggesting distinct starch properties suitable for food processing applications requiring high viscosity and rapid gelatinization, such as instant foods and convenience foods.

### 2.4. Correlation Analysis

**Figure 3:**
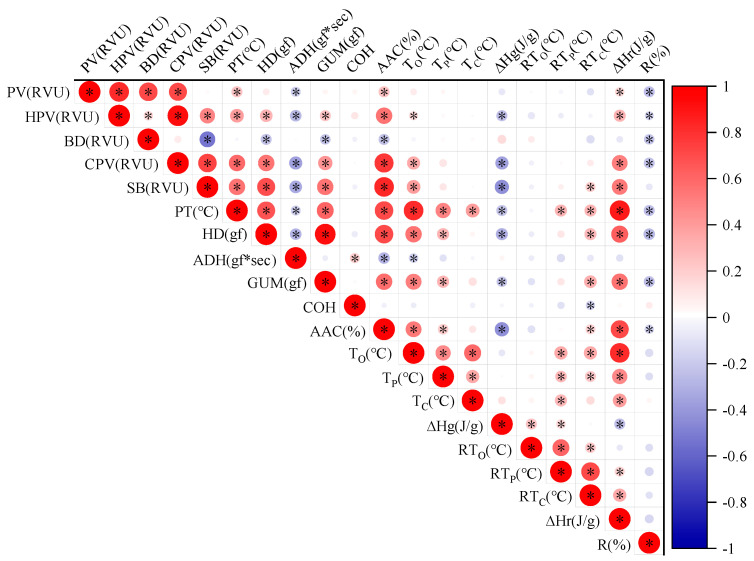
Correlation coefficients between rice CEQ traits. * indicate correlations are significant at *p* < 0.05.

The pair-wise correlation coefficients for each pair of the 20 traits are presented in Figure 3. Notably, AAC demonstrated significant correlations with all traits except COH, RTo (retrogradation onset temperature), and RTp (retrogradation peak temperature). Specifically, AAC exhibited negative correlations with BD, ADH, and ∆Hg, while positive correlations were found between AAC and HPV, CPV, SB, HD, GUM, ∆Hr, and R%. Among the pasting viscosity parameters, the majority of pairs displayed correlations, with only three pairs lacking statistical significance. Additionally, PT had a significant correlation with most thermal parameters and retrogradation properties.

### 2.5. GWAS Results

**Figure 4:**
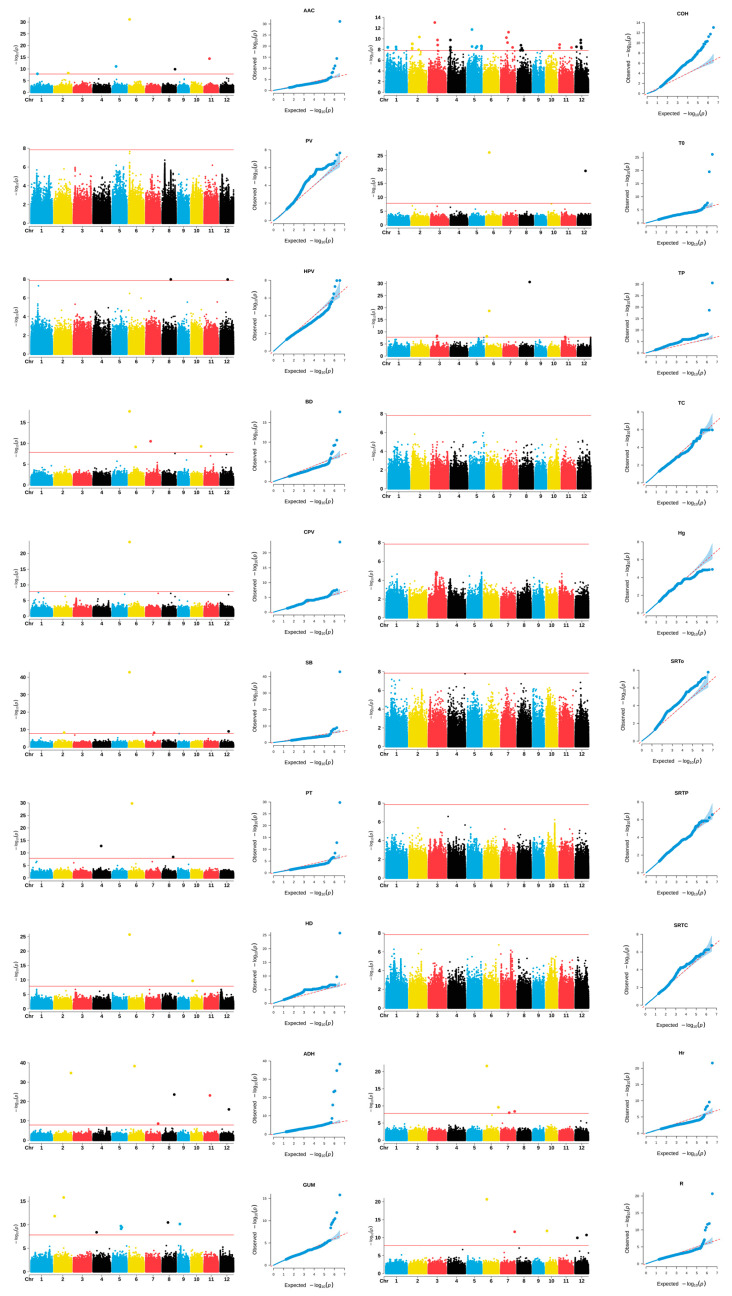
Manhattan (**left**) and quantile–quantile plots (**right**) of the genome-wide association study for quality traits of rice accessions. The points in the Manhattan plots indicate the −log10(*p*) values. The horizontal red line indicates the significant thresholds.

The fixed and random model circulating probability unification (FarmCPU) approaches were applied to conduct a GWAS for 20 CEQ traits. The marker trait association (MTA) regions were strictly defined by LD blocks of 183 kb left and right genomic regions of significant SNPs (SNP ± LD) for the whole panel, as previously calculated. Furthermore, the SNP with the lowest *p*-value was regarded as the lead SNP. In the Manhattan plot, a pronounced locus structure on the chromosome was observed for every trait. A total of 32 MTAs were identified in the whole panel (Table 2, Figure 4).

The *Wx* gene was identified significantly for AAC, BD, and GUM. In addition, we detected a locus (chr.6: 1620142) close to the *Wx* gene for SB. This SNP is in the gene encoding region (*LOC_Os06g03990*), where a substandard starch grain 6 (*SSG6*) gene encoding a protein homologous to aminotransferase is located. This protein plays a crucial role in regulating both the size and morphology of starch granules within endosperm cells [35]. The *SSIIa* gene was identified significantly for Tp and R. One locus (chr.7:28619594) was detected for both HPV and R, and the other locus (chr.7: 25202120) for AAC was very close to it.

### 2.6. Identification of Favorable Alleles in the Wx and SSIIa Genes

**Figure 5:**
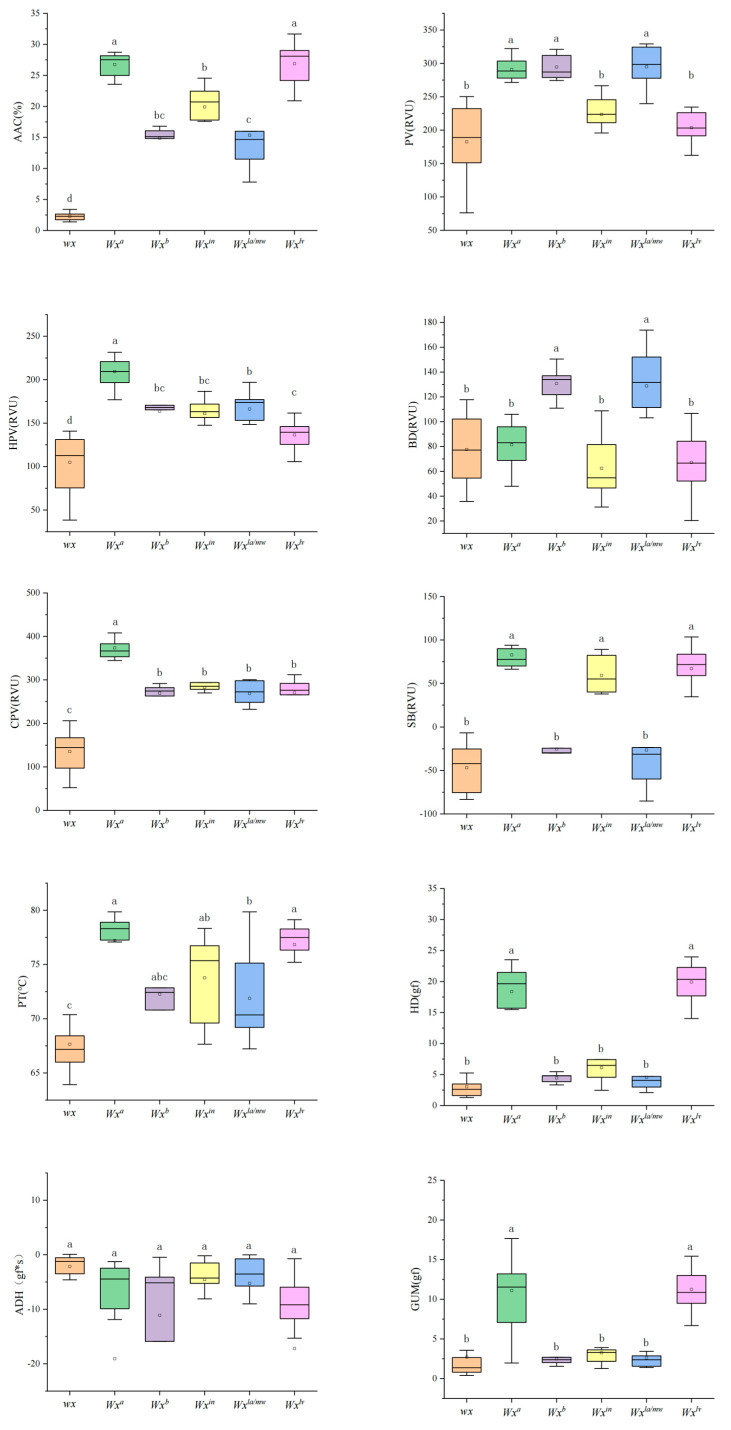

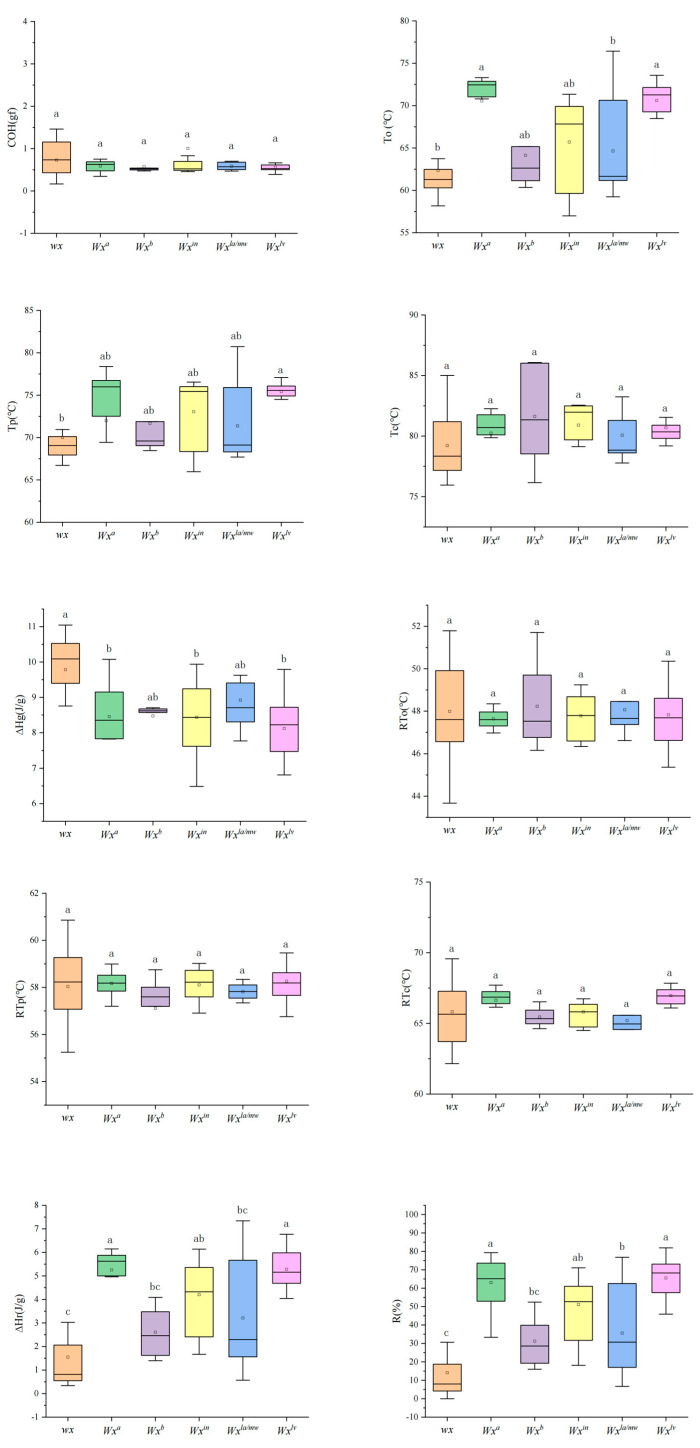
The distributions of quality traits in six panels, *wx* panel, *Wx^a^* panel, *Wx^b^* panel, *Wx^in^* panel, *Wx^la/mw^* panel, and *Wx^lv^* panel. Different letters indicate significant differences among means (*p* < 0.05).

The distribution and nucleotide diversity of various *Wx* alleles across all rice materials were analyzed. Analysis of nucleotide polymorphism in *Wx* revealed that the predominant *Wx* alleles were *wx*, *Wx^a^*, *Wx^b^*, *Wx^in^*, *Wx^la/mw^*, and *Wx^lv^* among all rice accessions [6,13,17]. These findings suggest that Shanlan rice is a valuable germplasm resource for high-quality rice breeding. The physicochemical properties of these materials were compared among different *Wx* alleles (Figure 5; Table S2). Notably, the average AAC of the six panels exhibited significant differences, with *Wx^lv^* having the highest AAC and *wx* the lowest (Figure 5). This finding aligns with previous studies [12]. Additionally, the pasting viscosity also differed significantly among allelic populations (Figure 5). Differences were also observed in textural properties such as HD and GUM. When it comes to thermal properties, however, there were only minor differences between panels (Figure 5).

**Figure 6:**
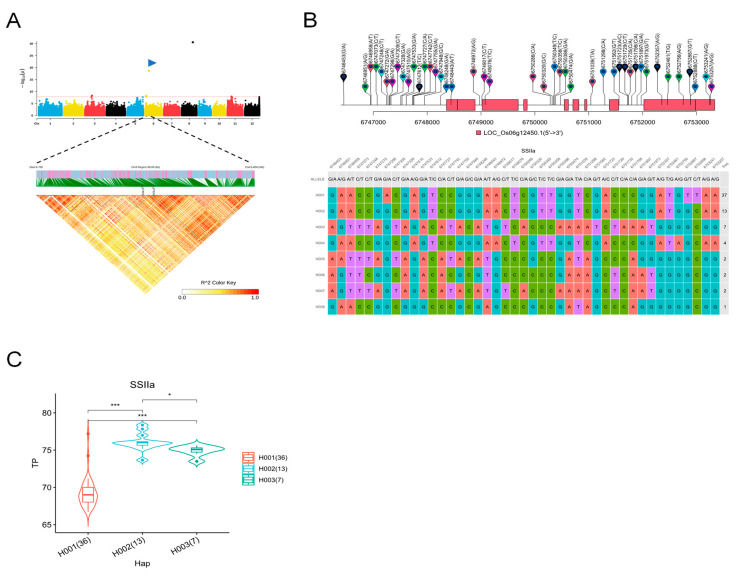
Identification of candidate genes for Tp in the whole panel. (**A**) High-density gene-based association analysis and LD heat map of local Manhattan map, around the peak on chromosome 6 highlighted with a blue triangle. (**B**) Based on 43 SNPs in all evaluated rice accessions, 8 haplotypes of *SSIIa* (*LOC_Os06g12450*) were identified. In the gene structure diagram of *LOC_Os06g12450*, the exon and UTR are indicated by the red frame; and the intron and intergenic regions are marked by black lines. (**C**) Comparison of Tp among accessions carrying different haplotypes of *SSIIa*, and haplotypes with fewer than 5 accessions are not shown. * and *** indicate correlations are significant at *p* < 0.05 and *p* < 0.001, respectively.

*SSIIa* was a significant MTA detected for multiple traits and was selected for high-density association and gene-based haplotype analysis. A total of 9439 SNPs were used for high-density association analysis of Tp in the 6.7 to 6.8 Mb region of chromosome 6 (Figure 6A). Eight haplotypes were detected in all germplasms, based on seventeen SNPs in the *SSIIa* promoter, six SNPs in the exon, three SNPs in the UTR, and seventeen SNPs in the intron (Figure 6B). Haplotype analysis showed that Tp in H001 was significantly lower than that in the other two haplotypes (Figure 6C).

### 2.7. Identification of Candidate Genes

**Figure 7:**
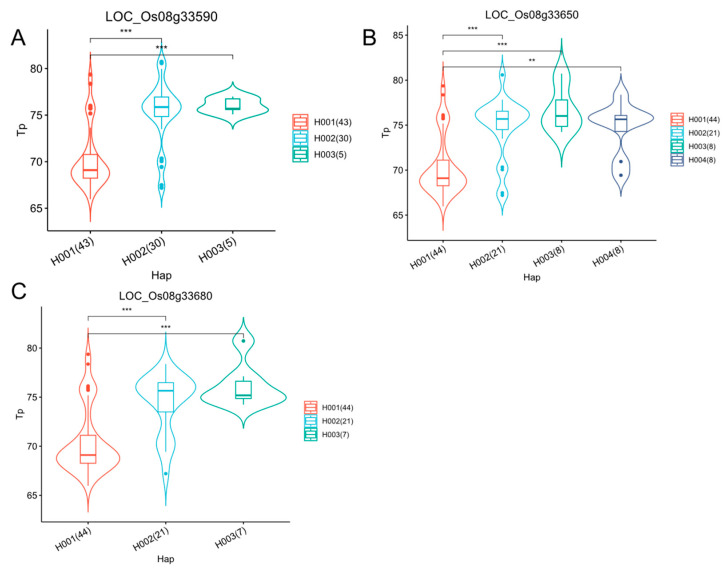
Haplotype analysis was conducted on three genes (*LOC_Os08g33590* (**A**), *LOC_Os08g33650* (**B**), *LOC_Os08g33680* (**C**)) that harbored nonsynonymous SNPs and UTR SNPs significantly associated with Tp. ** and *** indicate correlations are significant at *p* < 0.01 and *p* < 0.001, respectively.

To identify other candidate genes related to the CEQ traits in the remaining loci, we extracted all SNPs in the 50 kb left and right genomic regions of important MTAs (accounting for over 20% of the phenotypic variance explained). Four important MTAs were selected and used for gene-based association study with the mixed linear model (MLM) in Tassel 5.0. For the MTA S2_19767079 and MTA S5_17739626 for the GUM trait (Table 2), we detected two significant SNPs (*p* < 0.001) in the 19.71–19.81 Mb candidate region, and two significant SNPs (*p* < 0.001) in the 17.68–17.78 Mb candidate region. According to SNP information, they were all upstream and downstream SNPs (Tables S3 and S4). For MTA S12_16601981 for To, we detected two significant SNPs (*p* < 0.001) in the 16.55–16.65 Mb candidate region and they were both intergenic SNPs (Table S5). For MTA S8_20995138 for Tp, we detected 113 significant SNPs (*p* < 0.00006) in the 20.94–21.04 Mb candidate region and selected ten nonsynonymous SNPs and SNPs located on UTR (Table S6). These SNPs located within five genes (LOC_Os08g33590, LOC_Os08g33600, LOC_Os08g33610, LOC_Os08g33650, LOC_Os08g33680). Based on the gene annotations from the Rice Genome Annotation Project (RGAP), LOC_Os08g33600 and LOC_Os08g33610 were annotated as retrotransposon. Then we conducted haplotype analysis for the remaining three genes. Interestingly, for these genes, there were highly significant differences between Hap 1 and Hap 2/ Hap 3 at *p* < 0.001, and there were no significant differences between Hap 2 and Hap3 (Figure 7). Based on the LD analysis, we found that there was a substantial LD block region from 20973028 bp to 21116123 bp (Figure S2), which may be responsible for the similar haplotypes among these genes. Fine-mapping and identifying the candidate gene by transgenic experiment for this region may provide direct evidence to their association with Tp.

## 3. Discussion

### 3.1. Population Structure Analysis Indicates Shanlan Landrace Rice Is a Specific Rice Germplasm

Population structure analysis in this study showed that the 90 rice germplasms could be divided into *japonica* and *indica* subspecies when K = 2 was selected. This is true since the *indica* and *japonica* subspecies of *Oryza sativa* have a long-standing divergence that is both ancient and well-established [36]. Chen et al. [37] developed a set of SNPs and identified the similar subspecific differentiation and distinct geographic patterns within the *indica* and *japonica* germplasms. However, when K = 3 was selected, the *indica* group remained the same, while the *japonica* group was further divided into two subpopulations, namely GJ1 and GJ2. The GJ1 group only had seven accessions including three typical *japonica* cultivars, suggesting that the *japonica* Shanlan landraces had different characteristics from the modern *japonica* cultivars, while the *indica* Shanlan landraces are similar to modern *indica* cultivars. These results may raise interesting questions such as what is the origin of Shanlan rice and how did it evolve to the modern cultivars? Although Li et al. [5] suggested that the Shanlan rice in Hainan may have originated from Guangdong Province using SSR markers, these results may be challenged if using genome re-sequencing data, because they only used a few SSR markers in the analysis. However, together with the previous studies [2,3], we can conclude that both the *indica* and *japonica* subspecies were found in Shanlan landrace. The LD decay distance we calculated in our study for all germplasms was 183 kb, which was longer than that of a set of 809 indica rice accessions [38] and the 3 k rice population [39]. The difference in LD decay distance between our investigation and the previous ones may imply that Shanlan landrace rice has a lower genetic diversity, because these rice groups were produced through traditional slash-and-burn cultivation in the mountains [4].

### 3.2. Shanlan Landrace Rice Displayed Wide Diversity in Starch Physicochemical Properties

As rice yields increase, the cultivation of high-quality rice has become a prime objective for breeders. In general, CEQs are elusive traits that are much harder to select than other more obvious traits. Limited information on the diversity of cooking quality traits in the upland landrace rice has been available. Toosang et al. [40] examined grain quality traits of Thai highland glutinous rice landraces and found diversity in the cooking quality parameters. In this study, we comprehensively assessed the physicochemical properties of starch in Hainan Shanlan rice accessions, covering various aspects such as AAC and paste viscosity parameters, texture parameters, thermal properties, and retrogradation properties. These properties exhibited significant differences among different populations. In particular, the AAC values of the entire japonica rice population exhibited a notably higher coefficient of variation (CV, 76.30%). Correspondingly, their viscosity characteristics also displayed great diversity, which can be attributed to the existence of diverse *wx* alleles within this population. Conversely, the indica rice population demonstrated a lower coefficient of variation and exhibited more consistent characteristics. Feng et al. [41] conducted a comparative analysis of the quality traits of 635 rice samples from China, revealing significant disparities between the japonica and indica rice varieties. These findings align closely with the results of our study.

Previous research has reported significant correlations between AAC and various pasting viscosity parameters [42]. Our findings align with these results, as we observed a correlation between AAC and most pasting viscosity parameters. Additionally, previous studies have shown positive correlations between AAC and HD, CHEW, GUM, and COH, as well as a negative correlation with ADH [43,44], which is consistent with our findings. Hori et al. [45] found significant positive correlations between HD and PV, HPV, CPV, SB, and PT, as well as significant negative correlations between ADH and CPV, SB, and PT. Our results also demonstrate similar significant correlations. Although some correlations vary across different studies, likely due to the diversity of rice germplasms or various environmental conditions, our findings generally align with previous research. The consistent correlations between AAC, paste properties, and textural attributes may be attributed to their physicochemical and even genetic relationships. These relationships can be exploited by rice breeders in advanced breeding generations for trait selection.

### 3.3. Shanlan Landrace Rice Contains Many Favorable Wx Alleles

In this study, GWAS was used to analyze the CEQ traits, leading to the identification of 32 significant loci. Of these, 26 were novel loci (Table 1). Notably, the *Wx* locus emerged as a major MTA. These findings align with correlation analysis and previous reports [46]. Zhao et al. [47] performed association mapping on 83 *indica* and 170 *japonica* rice accessions using 210 SSR markers and identified 14 loci where the *Wx* gene exhibited significant associations with AAC, GC, and pasting viscosities. Yang et al. [26] conducted association mapping with 143 markers in nonwaxy rice and found that *Wx* was a major main-effect QTL for gel texture (including HD, ADH, and COH). Misra et al. [48] detected major effect genetic loci on the *Wx* gene for AAC and ADH using both single-locus GWAS and multi-locus GWAS in 236 indica accessions. Collectively, these findings suggest that the *Wx* gene plays a pivotal role in determining AAC, texture, and pasting viscosity.

This study focused on the comprehensive analysis of *Wx* alleles found in all the accessions, particularly the GJ group, which consists of accessions bearing the *wx*, *Wx^b^, Wx^in^,* and *Wx^la/mw^* haplotypes. Furthermore, group XI encompasses accessions with distinct *Wx* alleles, including *wx*, *Wx^a^*, *Wx^lv^*, *Wx^in^*, and *Wx^la/mw^*, each of which exhibits distinguishable characteristics. Notably, all accessions containing the *Wx^lv^* allele are categorized under group XI. Zhang et al. [12] mapped a specific allele of *Wx*, *Wx^lv^*, from a local indica rice variety. In this study, it was found that the rice with *Wx^lv^* showed high amylose content and low starch viscosity characteristics (Figure 3), which is in agreement with Zhang et al. [12]. It was proved that the *Wx^lv^* and *Wx* genes in wild rice have basically the same sequence and function, and belong to an ancestor gene in evolution. Interestingly, a small number of modern indica rice varieties were found to contain the *Wx^lv^* allele, while the majority of Shanlan landrace rice accessions contained the *Wx^lv^* allele. This fact may suggest that this allele in Shanlan rice received poor artificial selection. Another interesting finding is that *Wx^la/mw^* was reported as a rare allele in rice population [13,17]; however, this is not case in Shanlan landrace, since 13 accessions were found to contain this allele. The diverse *Wx* alleles found in Shanlan landrace rice provide rich gene resources for breeding new rice cultivars with improved CEQs.

### 3.4. New Loci Have Been Identified in Tp

The *SSIIa* gene is responsible for the elongation of short chains with degree of polymerization (DP) ≤ 12 (A chains) to B1 chains (13 ≤ DP ≤ 24) within the amylopectin cluster and has been found to be the main gene controlling GT [6]. *SSIIa* is the main gene controlling GT which is consistent with previous studies [49]. Furthermore, we examined all the genes in the genomic region of about 50 kb located in important MTAs (accounting for more than 20% of the phenotypic variation of the trait) from the Rice Genome Annotation Project (RGAP). Three genes related to Tp were screened out (*LOC_Os08g33590*, *LOC_Os08g33650*, *LOC_Os08g33680*). *OsbHLH38* (*LOC_Os08g33590*) is an alkaline helix-loop-helix transcription factor gene that is most important for ABA sensitivity. In addition, *OsbHLH38* influences abiotic stress tolerance in rice by mediating the expression of a large number of plant hormone transporter genes, transcription factor genes, and many downstream genes with different functions, including photosynthesis, redox homeostasis, and abiotic stress reactivity [50]. Further transgenic experiments are needed to reveal the association between these genes and Tp.

Molecular breeding by rational design is a cutting-edge strategy for rapid and precise crop improvement, leveraging known beneficial gene alleles and quantitative trait loci (QTLs) [51]. For instance, high-yield and high-quality elite cultivars have been developed by stacking multiple favorable alleles [52,53]. In summary, our results indicate that the Shanlan landrace rice contain wide genetic diversity in starch physicochemical properties derived from rich alleles of *Wx*. These alleles constitute a valuable resource for future genetic studies and rice improvement of rice grain quality.

## 4. Materials and Methods

### 4.1. Materials

A total of 84 Shanlan rice accessions were selected from the Hainan Province. In addition, three typical *japonica* rice cultivars, i.e., Nipponbare (Nip), Zhonghua 11 (ZH11), and Tainung 67 (TN67), and three typical *indica* rice cultivars, i.e., 9311, Guangluai4 (GLA4), and IR36 were used as references. All the rice accessions were planted and then harvested at the experimental farm of Sanya, China in 2022. After being air-dried, all the samples were stored at room temperature for a period of two and a half months. The rice grains were dehulled (Type THU, Satake Co., Tokyo, Japan), polished (Type TM05C, Satake Manufacturing, Suzhou, China), and then ground to flour (Cyclone Sample Mill, UDY Corporation, Fort Collins, CO, USA) to pass through a 100-mesh sieve.

### 4.2. DNA Extraction, Genotyping, and Linkage Disequilibrium

For each accession, two leaves were collected from a single plant at the tillering stage (one month after seedling transplantation), and genomic DNA was extracted from leaf samples using Plant DNA Mini Kits (Aidlab Biotech, Beijing, China). Sequencing libraries were generated using a Truseq Nano DNA HT Sample Preparation Kit (Illumina, San Diego, CA, USA) following the manufacturer’s recommendations. Separate index codes were added to each sample. The insert size of each library was approximately 350 bp. The Illumina HiSeq X10 platform was used to obtain the pair-ends of 150 bp reads, and the original sequence was further processed to remove adaptor-containing and low-quality reads. Library construction, sequencing, and sequence cleaning were all performed by Novogene Bioinformatics Technology Co., Ltd. (Beijing, China).

The reference genome was Nipponbare. GATK was used to call SNPs [54]. The mapping results were converted to the VCF format using SAMtools (version 0.1.18) [55]. SNPs with MAF ≥ 5% and missing rate ≤ 10% were retained, and 3,425,822 high-quality SNPs were finally obtained. The linkage disequilibrium (LD; R^2^) between pairs of markers was calculated using the software PopLDdecay version 3.42 [56]. When R^2^ declined to half of its maximum value, the distance across the chromosome was determined as the distance of LD decay [36].

### 4.3. Population Structure, Phylogenetic Tree Construction, and Kinship Analysis

The Tassel5.0 [57] was used to calculate the kinship (K) and principal component analysis (PCA). All SNPs were used in the calculation. A total of 93,302 independent SNPs across the whole genome determined by PLINK version 1.90 (window size 50, step size 10, R^2^ ≥ 0.1) [58] were used for population structure analysis by the Admixture version 1.3.0 [59].

### 4.4. Analysis of Starch Physicochemical Properties

AAC was measured using the iodine staining method described in Zhao et al. [60]. Each sample was replicated four times for reproducibility.

The pasting viscosities of rice flour were measured by Rapid Visco Analyser (Model 4500, Perten Instrument, Hägersten, Sweden) using its corresponding software program TCW3 (Thermocline for Windows 3). The viscosity parameters and pasting temperature (PT) were recorded or calculated from the same software. The unit of the viscosity was RVU (rapid viscosity unit). Each sample was replicated twice for reproducibility.

The aluminum cans with rice flour gels were sealed by Parafilm after the RVA analysis and stored at 4 °C for 24 h. Texture characteristics were measured by a texture analyzer (TA.XTC-18, Shanghai Bosin Industrial Development Co., Shanghai, China) using a standard two-cycle TPA program. A 5 mm diameter probe was used to compress the gel for 10 mm at 1 mm/s test speed. The hardness (HD, g), adhesiveness (ADH, g·s), gumminess (GUM, gf), and cohesiveness (COH) were derived from the software of the instrument.

The gelatinization and degradation characteristics were thoroughly analyzed using a Q20 differential scanning calorimeter (TA Instruments, New Castle, DE, USA). The method was according to Zhao et al. [60] with slight modifications. Initially, rice flour (2 mg with a moisture content of 12%) and water were weighed in a 1:3 weight ratio and subsequently sealed in an aluminum crucible. The mixture was then equilibrated at 4 °C for 24 h and at room temperature for an additional hour. After that, the crucible was placed in a DSC furnace and maintained at 30 °C for 1 min. Subsequently, the crucible was heated from 30 °C to 110 °C at a rate of 10 °C/min, with an empty crucible serving as a reference. The gelatinization parameters were derived from the software. After the initial measurements, the gelatinized samples from the DSC were kept at 4 °C for one week (placed in sample pans). Subsequently, the measurements were repeated to obtain the retrogradation parameters.

### 4.5. Genome-Wide Association Study

The total of 3,425,822 SNPs were selected for the GWAS analysis using fixed and random model circulating probability unification (FarmCPU) by the Genomic Association and Prediction Integrated Tool (GAPIT3) [61]. The first three principal components were used as covariates to capture the variance caused by population structure. The genome-wide significant thresholds of the GWAS (*p*-value = 2.92 × 10^−7^) were determined by 0.05/n (n is the number of markers). The Manhattan, QQ, and SNP-density plots for the GWAS were visualized using the R package CMplot version 3.1.3). The leading SNPs of each significant SNP cluster (in 200 kb) were selected to display the location of the MTAs.

### 4.6. Candidate Gene Analysis

To identify other candidate genes related to the CEQ traits in the remaining loci, we extracted all SNPs in the 50 kb left and right genomic regions of important MTAs (those accounting for over 20% of the phenotypic variance explained). Gene-based association analysis was conducted with the mixed linear model (MLM) in Tassel 5.0. The SNP types and gene annotations in the candidate region were analyzed. Finally, the candidate genes were determined based on the significant *p*-value, SNP information, and gene annotation.

### 4.7. Statistical Analysis

The calculations of means, standard deviation, and range of phenotypic data were performed using Excel. The results were presented as mean ± standard deviation (SD), in which all the measurements were accomplished at least in duplicate. Duncan’s multiple range test of ANOVA and correlation analysis were conducted using the SPSS 25.0 software (SPSS, Inc., Chicago, IL, USA).

## 5. Conclusions

The population structure and genetic diversity of Shanlan landrace rice were investigated with SNPs derived from the genome resequencing. Three subpopulations were derived from an analysis of population structure, GJ1, GJ2, and XI. Most japonica Shanlan rice belonged to GJ2 which was different from modern japonica cultivars, but most indica Shanlan rice were similar to the modern indica rice. There were considerable genetic variations for 20 CEQ traits in the population. We identified 32 MTAs in the whole panel, in which the *Wx*, *SSG6*, and *SSIIa* genes were the major candidate genes. These findings enhance our understanding of the genetic structure of Shanlan rice and richness of gene resources for the improvement of high-quality rice in current breeding programs.

## Figures and Tables

**Table 1:** Summary of genomic variants in rice populations.

SNPs	Number	InDels	Number
Total SNPs	11,266,589	Total Indels	1,635,574
SNPs in exon	2,619,968	Indels in exon	158,603
SNPs in intron	1,725,345	Indels in intron	379,545
SNPs in UTR5	84,864	Indels in UTR5	42,070
SNPs in UTR3	139,473	Indels in UTR3	51,556
SNPs in intergenic region	2,399,456	Indels in intergenic region	527,164
Upstream	2,182,885	Upstream	486,752
Downstream	1,735,090	Downstream	390,218
Non-synonymous SNPs	1,412,309	Splicing Indels	1971
Splicing SNPs	12,979	Stop-gain Indels	6580
Stop-gain SNPs	77,011	Stop-loss Indels	343
Stop-loss SNPs	4254	Frameshift Indels	95,147
		Non frameshift Indels	49,081

**Table 2:** Loci identified for quality traits of rice accessions by GWAS.

	SNP Position ^1^	*p*-Value	maf	Effect	PVE (%) ^2^	Candidate Gene
AAC	S1_13164701	1.17 × 10^−8^	0.48	0.04	0.32	
	S2_29156368	5.70 × 10^−9^	0.05	−0.04	3.87	
	S2_34721769	1.80 × 10^−35^	0.07	24.4	0.26	
	S5_7890154	8.14 × 10^−12^	0.1	0.04	1.38	
	S6_1779126	7.35 × 10^−32^	0.28	0.05	45.93	*Wx*
	S7_25202120	2.73 × 10^−9^	0.16	−6.76	0.08	
	S8_24746239	2.52 × 10^−24^	0.2	16.73	0.59	
	S11_10994972	3.95 × 10^−15^	0.09	0.05	5.4	
	S12_17949665	1.22 × 10^−16^	0.2	6.67	1.21	
BD	S6_1721569	2.09 × 10^−18^	0.12	−19.66	34.23	*Wx*
	S10_20833372	5.27 × 10^−10^	0.45	12.72	6.09	
GUM	S2_1491276	1.48 × 10^−12^	0.24	−1.67	10.54	
	S2_19767079	1.62 × 10^−16^	0.05	3.97	27.19	
	S4_7283069	4.07 × 10^−9^	0.09	1.45	18.39	
	S5_17739626	1.97 × 10^−10^	0.17	1.26	29.53	
	S5_18190039	7.49 × 10^−10^	0.26	−1	15.46	
	S6_1784985	1.87 × 10^−26^	0.37	7.25	18.47	*Wx*
	S8_11936414	3.32 × 10^−11^	0.24	1.43	4.22	
HPV	S7_28619594	4.13 × 10^−9^	0.11	0.97	0	
	S12_15381517	1.08 × 10^−8^	0.12	17.44	19.16	
R	S6_6745643	2.12 × 10^−21^	0.49	0.15	45.43	*SSIIa*
	S7_28619594	2.33 × 10^−12^	0.11	0.12	5.56	
	S10_2389557	1.33 × 10^−12^	0.27	0.1	9.45	
	S12_4781628	1.14 × 10^−10^	0.08	0.12	11.5	
SB	S6_1620142	1.31 × 10^−43^	0.5	−53.22	62.6	*SSG6*
	S7_16991812	6.79 × 10^−9^	0.15	21.03	6.77	
	S12_17341408	1.03 × 10^−9^	0.11	19.38	3.9	
To	S12_16601981	3.15 × 10^−20^	0.07	3.34	22.08	
Tp	S3_13207912	4.79 × 10^−9^	0.47	3.77	4.63	
	S6_6745643	2.15 × 10^−19^	0.5	2.89	45.91	*SSIIa*
	S8_20995138	2.43 × 10^−31^	0.49	−12.01	33.73	
	S11_7696066	1.25 × 10^−8^	0.47	−3.63	1.36	

^1^ The letter S indicates a SNP, the first number after S indicates the chromosome, and the second number indicates its physical position. ^2^ PVE: Phenotypic variance explained.

## Data Availability

Data are contained within the article and Supplementary Materials.

## Supplementary Materials

The supporting information can be downloaded at: https://www.mdpi.com/article/10.3390/ijms25063469/s1.
