# Supplementary material for: Population Structure and Genetic Diversity of Shanlan Landrace Rice for GWAS of Cooking and Eating Quality Traits
Source: Int J Mol Sci. 2024 Mar 19;25(6):3469. doi: 10.3390/ijms25063469 (PMC10970729; doi:10.3390/ijms25063469)
Supplement: Supplementary file 1 [file ijms-25-03469-s001.zip › ijms-2909305-supplementary.pdf]

Table S1. The diverse collection of 90 rice accession.

Code	Name	Population structure	*Wx* allele
SL01	Menjiading3	XI	*wx*
SL02	Baikezhan4	XI	Wx^{a}
SL03	Shenshuilian	XI	Wx^{lv}
SL04	menjiading2	XI	Wx^{a}
SL05	hualinuo2	GJ2	Wx^{in}
SL06	menjiaqin	GJ2	*wx*
SL07	menjianing	GJ2	*wx*
SL08	miaomumai2	XI	Wx^{a}
SL09	zhishouwan1	GJ2	Wx^{in}
SL10	zhishouwan2	GJ2	Wx^{b}
SL11	menmiaonong1	GJ2	$Wx^{la/mw}$
SL12	menmiaonong2	GJ2	$Wx^{la/mw}$
SL13	mennuo1	GJ2	*wx*
SL14	huanggu1	GJ2	*wx*
SL15	huanggu2	GJ2	$Wx^{la/mw}$
SL16	zhuangannuo	GJ2	*wx*
SL17	shanlannuo1	GJ2	*wx*
SL18	shanlannuo2	GJ2	Wx^{in}
SL19	shanlannuo3	GJ2	*wx*
SL20	hongkezidaoyan	XI	Wx^{lv}
SL21	guangtounuo	GJ2	*wx*
SL22	shanlan2	XI	$Wx^{la/mw}$
SL23	pole2	XI	Wx^{lv}
SL24	menjiaoyan	GJ2	Wx^{in}
SL25	menjiade2	XI	Wx^{lv}
SL26	baisilianggan	GJ2	$Wx^{la/mw}$
SL27	menjiaoyan1	GJ2	$Wx^{la/mw}$
SL28	shanlannuo3	GJ1	Wx^{in}
SL29	moyiqiu	GJ2	*wx*
SL30	heisidi1	GJ2	Wx^{b}
SL31	pozhan1	XI	Wx^{lv}
SL32	pozhan3	XI	Wx^{lv}
SL33	daohuangnuo	XI	$Wx^{la/mw}$
SL34	heimaonuo	GJ2	*wx*
SL35	zhongping1	GJ2	*wx*
SL36	zhongping2	GJ2	*wx*
SL37	zhongping3	GJ2	Wx^{in}
SL38	zhongping4	GJ2	Wx^{in}
SL39	zhongpingnian1	XI	Wx^{a}
SL40	zhongpingnian2	GJ2	*wx*

SL41	menkao2	XI	Wx^{in}
SL42	wuke3	XI	Wx^{lv}
SL43	wuke4	XI	Wx^{lv}
SL44	menjiafei1	XI	Wx^{a}
SL45	shanlan3	XI	Wx^{lv}
SL46	huangweishan2	XI	Wx^{lv}
SL47	menjiamei1	XI	Wx^{a}
SL48	menjiamei2	XI	Wx^{lv}
SL49	jialai1	GJ2	*wx*
SL50	kajialai2	GJ2	*wx*
SL51	gugu1	XI	Wx^{lv}
SL52	gugu3	GJ2	Wx^{b}
SL53	huangnuo3	XI	Wx^{a}
SL54	heidao1	XI	Wx^{a}
SL55	wanningnuo1	XI	Wx^{b}
SL56	shengsuanshanlan	GJ2	$Wx^{la/mw}$
SL57	menli	XI	Wx^{lv}
SL58	shanlannuo3	GJ2	$Wx^{la/mw}$
SL59	lvdao	GJ1	*wx*
SL60	shanlannuo3(hongmang)	XI	Wx^{in}
SL61	menjiati1	GJ2	$Wx^{la/mw}$
SL62	menjiati4	GJ1	Wx^{in}
SL63	pohe	XI	Wx^{lv}
SL64	shanlanzhumudao	GJ2	*wx*
SL65	lizhishanlan1	XI	Wx^{lv}
SL66	wuyuehong	XI	Wx^{lv}
SL67	fanjia	GJ2	*wx*
SL68	heimaozhan	GJ2	$Wx^{la/mw}$
SL69	shanlan1	GJ2	*wx*
SL70	shanlanuo2	GJ2	*wx*
SL71	heinuo	XI	*wx*
SL72	jinsi	XI	Wx^{a}
SL73	zhaonong	XI	Wx^{a}
SL74	shanlannuo2	XI	Wx^{lv}
SL75	shanlannuo3	XI	Wx^{lv}
SL76	shanlannuo4	GJ2	Wx^{b}
SL77	guangtounuo	XI	Wx^{a}
SL78	baishanlan	XI	*wx*
SL79	shanlanbainuo	XI	Wx^{lv}
SL80	shuangkehongmi	GJ2	$Wx^{la/mw}$
SL81	shanlanhongmi	XI	Wx^{lv}
SL82	shanlanheimi	XI	Wx^{a}
SL83	shanlandao1	XI	*wx*

SL84	shanlandao2	GJ1	$Wx^{la/mw}$
Nip	Nipponbare	GJ1	Wx^{b}
ZH11	Zhonghua11	GJ1	Wx^{b}
GLA4	Guangluai4	XI	Wx^{a}
IR36	IR36	XI	Wx^{a}
9311	9311	XI	Wx^{b}
TN67	Tainung67	GJ1	Wx^{b}

Table S2. Phenotypic variation in different among *Wx* alleles.

	wx		*Wx*^{a}		*Wx*^{b}		*Wx*^{in}		*Wx*$^{la/mw}$		*Wx*^{lv}	
	Means ± SD	Range	Means ± SD	Range	Means ± SD	Range	Means ± SD	Range	Means ± SD	Range	Means ± SD	Range
AAC (%)	2.31±0.6	1.6-3.2	26.6±2	23.6-28.7	16.1±2	14.8-17.8	19.9±4	17.6-24.6	15.4±5	11.0-26.4	26.7±6	20.9-31.7
PV（RVU）	182.5±52.3	76.2-250.3	290.8±26.1	229.3-344.7	294.7±18.5	274.4-321.1	223.5±25.7	171.0-266.5	295.0±30.2	239.7-329.2	203.7±29.4	174.8-234.5
HPV（RVU）	104.9±30.4	38.4-140.8	209.4±23.7	165.5-265.4	163.8±13.9	170.6-178.0	161.1±16.0	124.4-186.6	166.2±22.3	108.9-197.0	136.5±17.9	105.7-173.0
BD（RVU）	77.6±27.4	35.7-117.7	81.4±17.6	48.1-105.9	130.8±13.5	110.9-150.5	62.4±22.5	31.3-108.8	128.8±28.9	103.1-173.8	67.2±20.2	20.3-106.7
CPV（RVU）	135.7±40.2	52.3-206.3	373.6±35.5	323.3-474.2	269.4±18.8	236.9-291.5	282.6±29.1	269.8-293.8	268.5±50.4	232.0-300.4	270.8±35.6	265.3-333.8
SB（RVU）	-46.8±24.7	-83.3- -6.8	82.8±16.1	66.3-129.5	-25.2±11.1	-37.5- -21.7	59.1±18.6	37.8-89.1	-26.5±48.3	-85.1- -23.5	70.0±25.4	34.6-103.5
PT（℃）	67.2±1.8	63.9-71.2	77.2±2.8	70.0-79.9	72.3±3.0	68.1-77.2	73.8±3.8	67.7-78.3	71.9±3.8	67.2-79.9	76.8±2.1	70.4-79.1
HD（gf）	2.7±1.3	1.3-5.6	18.4±6.4	4.4-31.4	4.5±0.8	3.3-5.5	6.1±2.1	2.5-10.4	4.6±2.4	2.1-10.8	19.9±2.8	14.0-24.0
ADH（gf·s）	-2.2±2.2	-9.5- -0.1	-4.6±3.2	-11.9-1.4	-11.1±10.8	-30- -0.5	-4.5±7.0	-22-7.4	-5.3±6.8	-25.2-0	-17.2±23.1	-15.3- -4.1
GUM（gf）	2.2±2	0.4-8.1	11.1±5.1	2.0-17.7	2.5±0.7	1.6-3.8	3.3±1.5	1.3-7.2	2.6±1.3	1.4-6.2	11.3±2.7	6.7-17.8
COH（gf）	0.8±0.4	0.2-1.5	0.6±0.1	0.4-0.8	0.6±0.1	0.5-0.8	1.0±1.3	0.5-5.0	0.6±0.1	0.5-0.7	0.6±0.1	0.4-0.9
To（℃）	61.2±1.7	58.2-65.7	72.3±0.8	70.8-73.3	64.1±4.0	60.4-71.4	65.7±5.2	57.0-71.3	64.7±5.7	59.2-76.4	70.6±2.6	68.5-73.6
Tp（℃）	69.2±1.6	66.7-74.2	75.1±3.2	67.2-78.4	71.7±4.0	68.5-79.4	73.0±4.0	66.0-76.6	71.4±4.4	67.7-80.7	75.4±2.3	73.5-77.8
Tc（℃）	78.8±2.5	76.0-85.0	81.3±1.1	79.9-84.1	81.6±4.0	76.2-86.1	80.9±2.1	79.1-82.6	80.1±2.3	77.8-83.2	80.7±1.8	77.9-82.9
ΔHg（J/g）	10.1±0.6	8.8-11.0	8.7±0.7	7.8-10.1	8.5±0.3	7.8-8.7	8.4±1.1	6.5-9.9	8.9±0.8	7.8-9.6	8.1±0.9	5.9-9.8
RTo（℃）	48±2.2	43.7-51.8	47.6±0.4	47.0-48.4	48.2±2.0	46.2-47.7	47.8±1.0	46.3-49.2	48.1±1.3	46.6-48.9	47.8±1.4	45.4-51.4
RTp（℃）	58±1.4	55.3-60.9	58.2±0.4	57.2-59.0	57.1±1.6	57.1-58.8	58.1±0.7	56.9-59.0	57.8±0.5	56.8-58.9	58.3±1.1	56.1-59.5
RTc（℃）	65.4±2	62.2-69.6	66.9±0.8	66.2-68.3	65.5±0.7	64.6-66.5	65.8±1.9	64.5-66.7	65.2±2.0	64.6-67.8	67.0±1.5	63.5-72.2
ΔHr（J/g）	1.1±0.8	0.3-3.0	5.7±0.6	5.0-6.3	2.6±1.0	1.4-4.1	4.2±1.5	1.7-6.1	3.2±2.2	0.6-7.3	5.3±0.9	4.0-6.8
R（%）	11.5±8.5	3.5-36	59.2±14	33.4-79.3	37.5±13.7	19.3-53.8	46.3±15.5	18.1-71.0	35.6±22.9	6.7-76.8	66.8±9.5	45.9-81.9

Table S3. The SNP information in the 19.71–19.81 Mb candidate region for GUM.

MSU ID	Chromosome	SNP location	Reference	Alternative	Variation type	Associated signal in GWAS (*p* value)*
LOC_Os02g33250, LOC_Os02g33260(dist=501)	Chr2	19767079	A	G	upstream	7.44E-06
LOC_Os02g33250, LOC_Os02g33260(dist=499)	Chr2	19767081	T	G	upstream	6.69E-04

*The *p*-value was calculated by Tassel5 with MLM. SNPs with *p*-value less than 0.001 were listed.

Table S4. The SNP information in the 17.68–17.78 Mb candidate region for GUM.

MSU ID	Chromosome	SNP location	Reference	Alternative	Variation type	Associated signal in GWAS (*p* value)*
LOC_Os05g30620(dist=1108)	Chr5	17739626	A	G	downstream	3.54E-05
LOC_Os05g30560(dist=950)	Chr5	17708858	T	C	upstream	5.01E-04

*The *p*-value was calculated by Tassel5 with MLM. SNPs with *p*-value less than 0.001 were listed.

Table S5. The SNP information in the 16.55–16.65 Mb candidate region for To.

MSU ID	Chromosome	SNP location	Reference	Alternative	Variation type	Associated signal in GWAS (*p* value)*
LOC_Os12g28110(dist=3051), LOC_Os12g28137(dist=14770)	Chr12	16601969	G	T	intergenic	2.46E-04
LOC_Os12g28110(dist=3063), LOC_Os12g28137(dist=14758)	Chr12	16601981	G	A	intergenic	2.46E-04

*The *p*-value was calculated by Tassel5 with MLM. SNPs with *p*-value less than 0.001 were listed.

Table S6. The SNP information in the 20.94–21.04 Mb candidate region for Tp.

MSU ID	Chromosome	SNP location	Reference	Alternative	Variation type	Associated signal in GWAS *p* value)*
LOC_Os08g33610(dist=7172),LOC_Os08g33620(dist=4474)	Chr8	20992803	T	C	intergenic	2.28E-06
LOC_Os08g33620(dist=1733)	Chr8	20995544	G	A	upstream	2.28E-06
LOC_Os08g33610(dist=9491),LOC_Os08g33620(dist=2155)	Chr8	20995122	A	G	intergenic	5.60E-06
LOC_Os08g33610(dist=9507),LOC_Os08g33620(dist=2139)	Chr8	20995138	T	C	intergenic	5.60E-06
LOC_Os08g33620(dist=1288)	Chr8	20995989	A	C	upstream	2.80E-05
LOC_Os08g33620(dist=1278)	Chr8	20995999	T	C	upstream	2.80E-05
LOC_Os08g33590(dist=1692)	Chr8	20972567	A	G	downstream	3.07E-05
LOC_Os08g33690	Chr8	21037004	A	G	exonic, synonymous SNV	3.13E-05
LOC_Os08g33590(LOC_Os08g33590.1:c.-188G>C)	Chr8	20977227	C	G	UTR5	3.34E-05
LOC_Os08g33700	Chr8	21043296	T	C	exonic, synonymous SNV	3.34E-05
LOC_Os08g33660(dist=525)	Chr8	21021988	A	G	upstream	4.78E-05
LOC_Os08g33620(dist=1811)	Chr8	20995466	A	G	upstream	5.18E-05
LOC_Os08g33620,LOC_Os08g33630(dist=1662)	Chr8	20998398	A	G	downstream	5.29E-05
LOC_Os08g33590(dist=246)	Chr8	20977500	C	G	upstream	5.35E-05
LOC_Os08g33610(dist=886)	Chr8	20986517	A	T	upstream	5.35E-05
LOC_Os08g33610(dist=898)	Chr8	20986529	C	T	upstream	5.35E-05
LOC_Os08g33610(dist=4207),LOC_Os08g33620(dist=7439)	Chr8	20989838	T	C	intergenic	5.37E-05
LOC_Os08g33630(dist=662)	Chr8	21004608	A	G	upstream	5.37E-05
LOC_Os08g33630(dist=667)	Chr8	21004613	C	A	upstream	5.37E-05
LOC_Os08g33640(dist=952)	Chr8	21009762	A	C	upstream	5.37E-05
LOC_Os08g33640(dist=743)	Chr8	21009971	G	A	upstream	5.37E-05
LOC_Os08g33680(LOC_Os08g33680.1:c.-13C>G)	Chr8	21036007	G	C	UTR5	5.37E-05
LOC_Os08g33610	Chr8	20985222	T	A	intronic	5.41E-05
LOC_Os08g33610	Chr8	20985159	T	C	intronic	5.43E-05
LOC_Os08g33610	Chr8	20985191	C	T	intronic	5.43E-05
LOC_Os08g33670(dist=476)	Chr8	21029435	A	G	upstream	5.69E-05
LOC_Os08g33670(dist=477)	Chr8	21029436	G	A	upstream	5.69E-05
LOC_Os08g33670(dist=543)	Chr8	21029502	T	C	upstream	5.69E-05
LOC_Os08g33650	Chr8	21017560	T	G	intronic	5.70E-05
LOC_Os08g33690(dist=2167),LOC_Os08g33700(dist=3559)	Chr8	21039444	A	C	intergenic	5.74E-05
LOC_Os08g33690(dist=2480),LOC_Os08g33700(dist=3246)	Chr8	21039757	T	C	intergenic	5.74E-05
LOC_Os08g33690(dist=2519),LOC_Os08g33700(dist=3207)	Chr8	21039796	C	T	intergenic	5.74E-05
LOC_Os08g33590(dist=1511)	Chr8	20978765	A	C	upstream	5.77E-05

LOC_Os08g33690	Chr8	21036767	A	G	exonic, synonymous SNV	5.77E-05
LOC_Os08g33670(dist=2240),LOC_Os08g33680(dist=2979)	Chr8	21031199	A	G	intergenic	5.79E-05
LOC_Os08g33610(dist=5933),LOC_Os08g33620(dist=5713)	Chr8	20991564	T	C	intergenic	5.81E-05
LOC_Os08g33610(dist=6070),LOC_Os08g33620(dist=5576)	Chr8	20991701	G	T	intergenic	5.81E-05
LOC_Os08g33650(LOC_Os08g33650.1:c.*117A>G)	Chr8	21015720	A	G	UTR3	5.81E-05
LOC_Os08g33630(dist=449)	Chr8	21004395	T	C	upstream	5.84E-05
LOC_Os08g33630(dist=458)	Chr8	21004404	G	A	upstream	5.84E-05
LOC_Os08g33700(dist=1499)	Chr8	21041504	G	A	upstream	5.90E-05
LOC_Os08g33600	Chr8	20983484	A	G	exonic, synonymous SNV	5.93E-05
LOC_Os08g33690(dist=1001)	Chr8	21038278	T	G	downstream	5.93E-05
LOC_Os08g33600 (retrotransposon protein)	Chr8	20983863	A	G	exonic, nonsynonymous SNV	5.95E-05
LOC_Os08g33600 (retrotransposon protein)	Chr8	20984209	G	A	exonic, nonsynonymous SNV	5.95E-05
LOC_Os08g33630(dist=316)	Chr8	21004262	T	C	upstream	5.95E-05
LOC_Os08g33630(dist=332)	Chr8	21004278	C	T	upstream	5.95E-05
LOC_Os08g33630(dist=335)	Chr8	21004281	G	T	upstream	5.95E-05
LOC_Os08g33630(dist=351)	Chr8	21004297	T	G	upstream	5.95E-05
LOC_Os08g33630(dist=388)	Chr8	21004334	G	A	upstream	5.95E-05
LOC_Os08g33670(dist=1797)	Chr8	21023747	T	A	downstream	5.95E-05
LOC_Os08g33670	Chr8	21028183	A	C	intronic	5.95E-05
LOC_Os08g33670(dist=801)	Chr8	21029760	T	C	upstream	5.95E-05
LOC_Os08g33590(dist=1601)	Chr8	20978855	A	G	upstream	5.98E-05
LOC_Os08g33680(dist=688)	Chr8	21033490	A	C	downstream	5.98E-05
LOC_Os08g33630(dist=3999),LOC_Os08g33640(dist=2769)	Chr8	21007945	G	A	intergenic	5.98E-05
LOC_Os08g33630(dist=4010),LOC_Os08g33640(dist=2758)	Chr8	21007956	A	G	intergenic	5.98E-05
LOC_Os08g33680(dist=1370)	Chr8	21032808	G	A	downstream	5.98E-05
LOC_Os08g33680(dist=1366)	Chr8	21032812	C	G	downstream	5.98E-05
LOC_Os08g33680(dist=1350)	Chr8	21032828	A	G	downstream	5.98E-05
LOC_Os08g33680	Chr8	21034712	T	C	exonic, synonymous SNV	5.98E-05
LOC_Os08g33700(dist=1312)	Chr8	21041691	G	C	upstream	5.98E-05
LOC_Os08g33620,LOC_Os08g33630(dist=1648)	Chr8	20998412	T	C	downstream	5.98E-05
LOC_Os08g33620,LOC_Os08g33630(dist=1631)	Chr8	20998429	A	G	downstream	5.98E-05
LOC_Os08g33680(dist=1944)	Chr8	21032234	A	G	downstream	5.98E-05

LOC_Os08g33680	Chr8	21035183	G	A	intronic	5.98E-05
LOC_Os08g33670	Chr8	21027338	C	G	exonic, synonymous SNV	5.98E-05
LOC_Os08g33540	Chr8	20945875	T	A	intronic	5.98E-05
LOC_Os08g33540	Chr8	20945910	C	A	intronic	5.98E-05
LOC_Os08g33540	Chr8	20946268	C	T	intronic	5.98E-05
LOC_Os08g33540	Chr8	20946353	T	G	intronic	5.98E-05
LOC_Os08g33540,LOC_Os08g33550(dist=889)	Chr8	20948070	A	G	upstream	5.98E-05
LOC_Os08g33580(dist=3739),LOC_Os08g33590(dist=3158)	Chr8	20971101	T	C	intergenic	5.98E-05
LOC_Os08g33590(dist=1616)	Chr8	20972643	A	G	downstream	5.98E-05
LOC_Os08g33590	Chr8	20976317	G	A	intronic	5.98E-05
LOC_Os08g33590(dist=361)	Chr8	20977615	T	C	upstream	5.98E-05
LOC_Os08g33590(dist=539)	Chr8	20977793	A	T	upstream	5.98E-05
LOC_Os08g33590(dist=581)	Chr8	20977835	G	A	upstream	5.98E-05
LOC_Os08g33590(dist=1127)	Chr8	20978381	T	A	upstream	5.98E-05
LOC_Os08g33590(dist=1310)	Chr8	20978564	A	G	upstream	5.98E-05
LOC_Os08g33590(dist=1366)	Chr8	20978620	C	T	upstream	5.98E-05
LOC_Os08g33590(dist=1989)	Chr8	20979243	T	A	upstream	5.98E-05
LOC_Os08g33590(dist=2215),LOC_Os08g33600(dist=3227)	Chr8	20979469	G	A	intergenic	5.98E-05
LOC_Os08g33590(dist=2261),LOC_Os08g33600(dist=3181)	Chr8	20979515	C	A	intergenic	5.98E-05
LOC_Os08g33590(dist=2265),LOC_Os08g33600(dist=3177)	Chr8	20979519	A	T	intergenic	5.98E-05
LOC_Os08g33590(dist=2418),LOC_Os08g33600(dist=3024)	Chr8	20979672	G	A	intergenic	5.98E-05
LOC_Os08g33590(dist=2422),LOC_Os08g33600(dist=3020)	Chr8	20979676	G	A	intergenic	5.98E-05
LOC_Os08g33590(dist=2433),LOC_Os08g33600(dist=3009)	Chr8	20979687	G	A	intergenic	5.98E-05
LOC_Os08g33590(dist=2754),LOC_Os08g33600(dist=2688)	Chr8	20980008	C	T	intergenic	5.98E-05
LOC_Os08g33590(dist=2770),LOC_Os08g33600(dist=2672)	Chr8	20980024	T	A	intergenic	5.98E-05
LOC_Os08g33590(dist=3326),LOC_Os08g33600(dist=2116)	Chr8	20980580	T	G	intergenic	5.98E-05
LOC_Os08g33600(dist=1937)	Chr8	20980759	A	T	upstream	5.98E-05
LOC_Os08g33600(dist=1604)	Chr8	20981092	A	T	upstream	5.98E-05
LOC_Os08g33600(dist=1525)	Chr8	20981171	T	C	upstream	5.98E-05
LOC_Os08g33600(dist=1022)	Chr8	20981674	G	A	upstream	5.98E-05
LOC_Os08g33600(dist=328)	Chr8	20982368	T	G	upstream	5.98E-05
LOC_Os08g33600(dist=125)	Chr8	20982571	T	C	upstream	5.98E-05
LOC_Os08g33600	Chr8	20982782	A	G	exonic, synonymous SNV	5.98E-05
LOC_Os08g33600 (retrotransposon protein)	Chr8	20982813	A	G	exonic, nonsynonymous SNV	5.98E-05

LOC_Os08g33600 (retrotransposon protein)	Chr8	20982841	G	A	exonic, nonsynonymous SNV	5.98E-05
LOC_Os08g33600	Chr8	20982944	G	A	exonic, synonymous SNV	5.98E-05
LOC_Os08g33600 (retrotransposon protein)	Chr8	20983039	T	C	exonic, nonsynonymous SNV	5.98E-05
LOC_Os08g33600	Chr8	20983069	C	T	intronic	5.98E-05
LOC_Os08g33600	Chr8	20983080	C	T	intronic	5.98E-05
LOC_Os08g33600	Chr8	20983694	T	G	exonic, synonymous SNV	5.98E-05
LOC_Os08g33600,LOC_Os08g33610(dist=219)	Chr8	20984356	A	G	downstream	5.98E-05
LOC_Os08g33600,LOC_Os08g33610(dist=208)	Chr8	20984367	C	A	downstream	5.98E-05
LOC_Os08g33600,LOC_Os08g33610(dist=51)	Chr8	20984524	G	A	downstream	5.98E-05
LOC_Os08g33610 (retrotransposon protein)	Chr8	20984627	C	T	exonic, nonsynonymous SNV	5.98E-05
LOC_Os08g33610	Chr8	20984686	A	G	exonic, synonymous SNV	5.98E-05
LOC_Os08g33610	Chr8	20984938	G	A	exonic, synonymous SNV	5.98E-05
LOC_Os08g33610	Chr8	20984989	G	A	exonic, synonymous SNV	5.98E-05
LOC_Os08g33610 (retrotransposon protein)	Chr8	20985502	G	A	exonic, nonsynonymous SNV	5.98E-05

*The *p*-value was calculated by Tassel5 with MLM. SNPs with *p*-value less than 0.00006 were listed. The significant nonsynonymous SNPs or SNPs located on UTR were highlighted in yellow background.

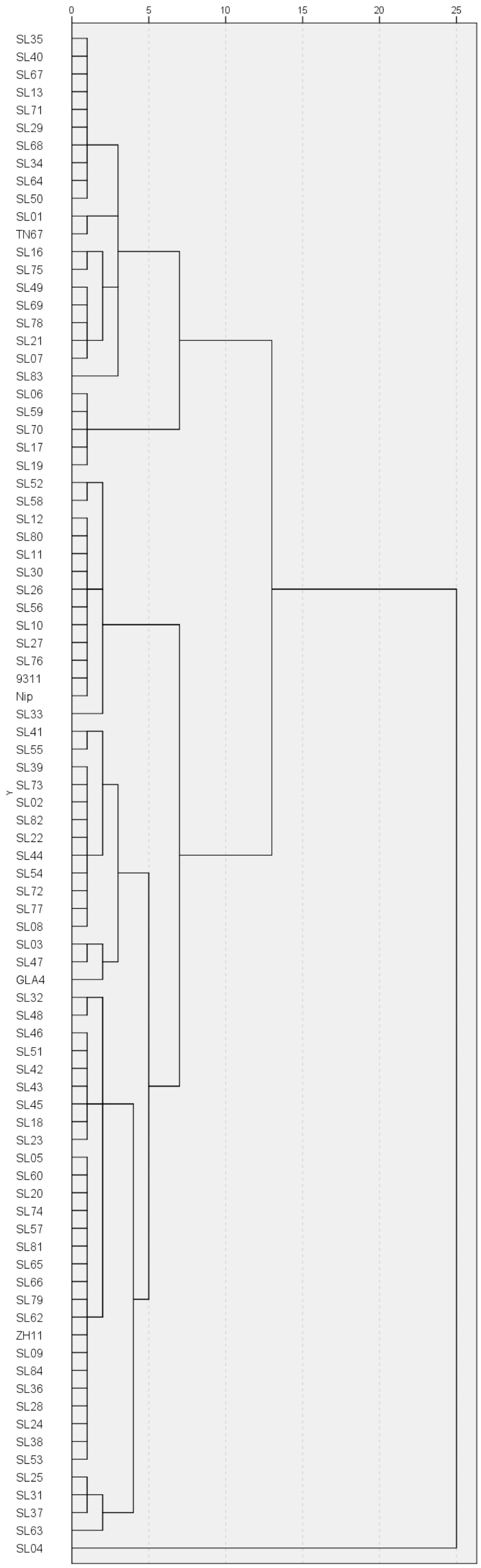

Figure S1. Cluster diagram of quality traits of different rice varieties.

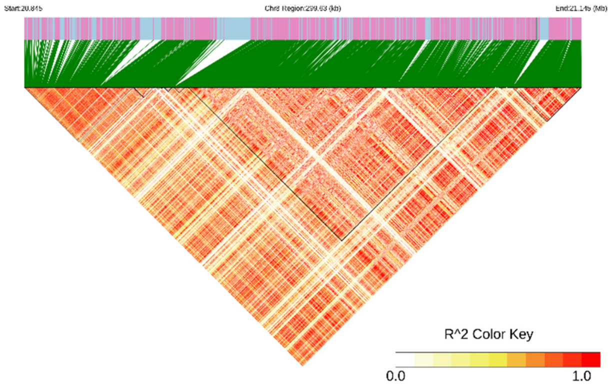

Figure S2. Identification of LD block in chromosome 8 for peak gelatinization temperature (Tp).
